# Tick burden in *Bos taurus* cattle and its relationship with heat stress in three agroecological zones in the tropics of Colombia

**DOI:** 10.1186/s13071-019-3319-9

**Published:** 2019-02-07

**Authors:** Juan Felipe Rocha, Rodrigo Martínez, Nicolas López-Villalobos, Steve Todd Morris

**Affiliations:** 1 Corporación Colombiana de Investigación Agropecuaria - Agrosavia, Km 14 Vía Mosquera, Bogotá, Cundinamarca 250047 Colombia; 2 School of Agriculture and Environment, Massey University, Palmerston North, 4474 New Zealand

**Keywords:** Tick burden, Adaptability index, Temperature humidity index, Creole cattle, Tropics, *Bos taurus*, Romosinuano, Costeño con Cuernos, San Martinero, Blanco Orejinegro

## Abstract

**Background:**

Ticks have a negative effect on dairy and beef cattle production systems around the world, with the concomitant risk they represent for the transmission of some important infectious diseases. Colombian cattle breeds are distributed across different agroecological regions and are exposed to different environmental challenges. In humid and warmer climates such as those from the tropics, tick burden and heat stress are important factors that can compromise livestock performance. The aim of this study was to characterize tick burden in four Colombian cattle breeds and evaluate the relationship between heat stress and tick burden in *Bos taurus* cattle under tropical conditions. Tick counting was conducted in 1332 cattle from Romosinuano (ROMO), Costeño con Cuernos (CCC), San Martinero (SM) and Blanco Orejinegro (BON) breeds, located in the Caribbean, Orinoquia and Andean regions. Vital signs and environmental variables were taken to calculate an adaptability index (AI) and a temperature humidity index (THI). An AI < 2 indicates maximum adaptability while an AI ≥ 2 indicates a state of lower adaptability. In beef cattle, productivity starts to be affected by heat stress when environmental conditions allow an estimation of a THI > 75.

**Results:**

Results showed a differing distribution of ticks on the body of individuals that varied according to the agroecological region. There was a significant effect of breed, sex, family, age and live weight on cattle tick burden. The lowest tick burden was observed in the ROMO breed (12.8 ± 2.6), while the highest tick burden was observed in CCC (31.8 ± 2.3), which were located in the same agroecological region. SM and ROMO animals with an AI > 2 had a higher tick burden than their counterparts that had an AI < 2.

**Conclusions:**

Cattle breed, sex, age and live weight affect the tick burden in *Bos taurus* Colombian cattle breeds. The tick burden is higher in cattle with lower adaptability to heat stress. Moreover, it decreases as heat stress levels increase in a tropical environment. The interaction between tick burden and environmental heat stress can be affected by characteristics of the agroecological region itself, the breed and the genetic resistance of the individual tick, as well as the thermal adaptability of cattle.

## Background

Ticks are bloodsucking ectoparasites that live by feeding on the blood of different companion animals, wildlife and livestock species. Ticks are particularly important in warmer, wetter habitats where they can affect agricultural production if not adequately controlled [1]. *Rhipicephalus* (*Boophilus*) *microplus* is an economically important tick widely distributed in the southern hemisphere and in the southern states of the USA, and acts as a vector for the transmission of babesiosis and anaplasmosis [2]. *Rhipicephalus microplus* is the tick with the highest negative economic impact for the beef and dairy cattle industries of Central and South America, and Australia [3, 4]. In Colombia, *R. microplus* has been reported in several departments across the Caribbean, the Andean and the Orinoquia regions [5].

Economic losses in infested cattle are due to skin damage, treatment costs, losses associated with the transmission of tick-borne diseases, and the direct negative effect on animal productivity [4, 6]. In *Bos taurus* cattle, each engorging female tick is responsible for the average loss of 1.37 ± 0.25 g of body weight per individual, while in *B. taurus* × *B. indicus* crosses, this value has been estimated as 1.18 ± 0.21 g/engorging tick. This body weight loss is not only due to the blood loss caused by ticks, but also due to appetite suppression and reduced feed intake, which may account for approximately 65% of the body weight depression. Moreover, alterations of metabolic parameters and impaired digestion by interference with normal abomasal outflow found in experimentally infested cattle indicate that the effect of blood loss on productivity only explains about 25% of the total bodyweight loss [6].

Climate is one of the main factors controlling tick distribution due to its regulatory influence on the tick life-cycle [7]. Changes in environmental conditions may have a positive or negative effect on tick burdens and on the physiological heat regulation mechanisms of infested cattle [8]. Several indices have been developed to monitor the heat stress status in different livestock species. The temperature-humidity index (THI) combines the effects of air temperature and humidity associated with the level of thermal stress [9]. Other indices such as Benezra’s coefficient of adaptability (AI) [10] or the Iberia heat tolerance test [11] account for vital signs in animals such as rectal temperature and respiratory rate.

Colombian cattle breeds were brought to the Americas more than 500 years ago. These breeds have an optimal reproductive performance [12], high meat quality [13] and have a particular resistance to diseases such as brucellosis [14]. The distribution of these breeds is affected by the extremely diverse agroecological conditions found in Colombia. Several studies have shown that besides the climate and habitat type, other factors such as the breed, age and sex of the animals also have a significant effect on the tick burden in different cattle populations [8, 15–18]. In Colombia, the available literature on cattle ticks addresses the biological and ecological aspects of these parasites [4] and its geographical distribution [5]. However, only a few studies have shown the tick burden on cattle kept under natural conditions, and these did not account for effects such as breed, age and sex of the animals or climate variables [19]. Therefore, and in view of the paucity of data on factors affecting tick infestations, the aim of this experiment was to characterize the tick burdens in Colombian cattle breeds and to evaluate the relationship between tick infestation levels and heat adaptability, using both the AI and the THI, under tropical conditions.

## Methods

### Study site and cattle breeds

**Fig. 1:**
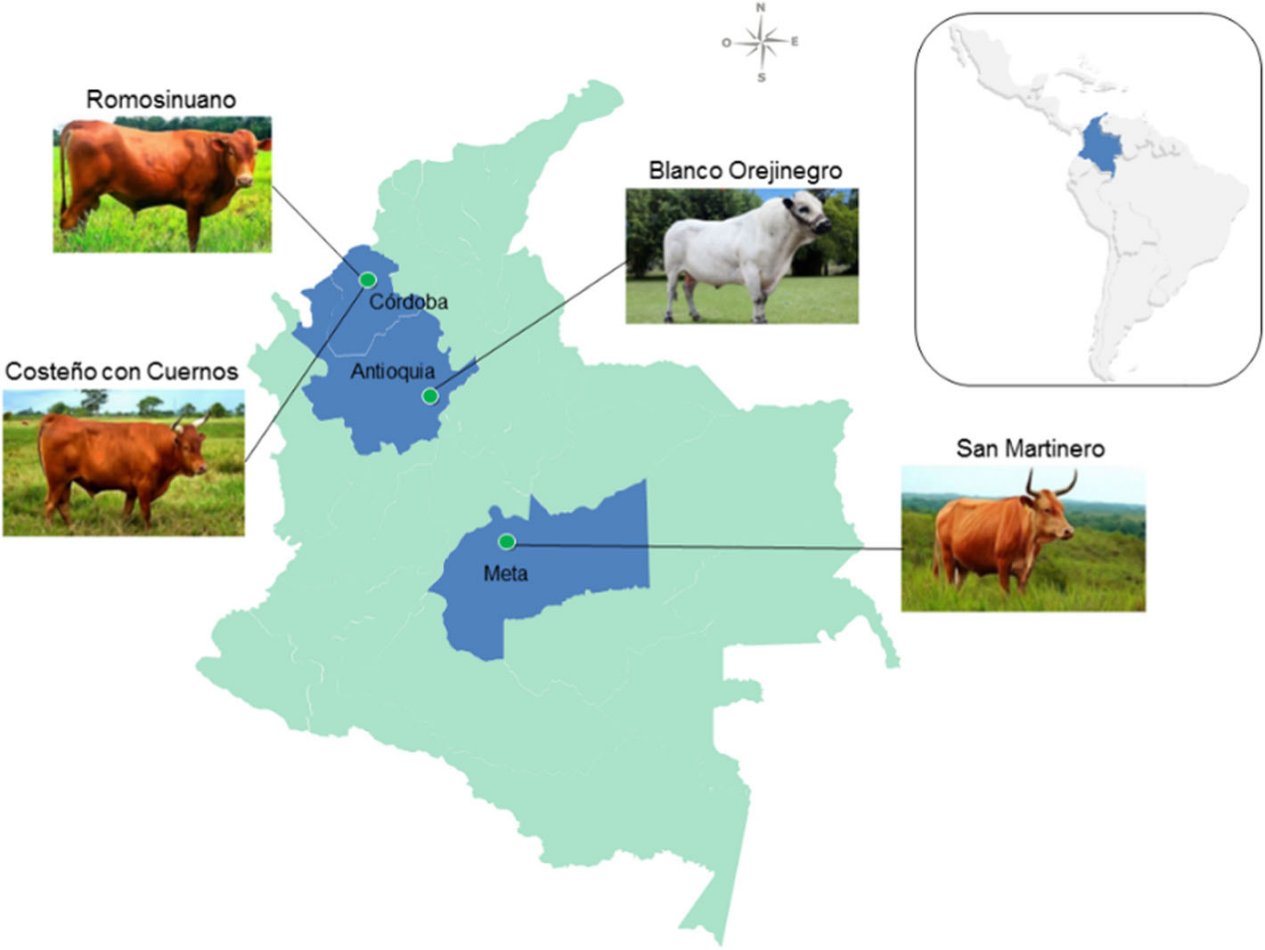
Geographical location of four Colombian cattle breeds included in this study. Dark green dots represent the agroecological zones of the experimental stations where each breed is kept. Highlighted regions in blue represent the departments of Cordoba, Antioquia and Meta in Colombia

The study was conducted at three experimental stations of Corporación Colombiana de Investigación Agropecuaria - Agrosavia and included four different Colombian *Bos taurus* cattle breeds. Figure 1 shows the geographical location for each breed. Blanco Orejinegro breed (BON; *n* = 276) were maintained in the experimental station El Nus, located in San Roque, Antioquia. These were fed with a mixture of *Brachiaria decumbens* (signal grass), *Brachiaria brizantha* (palisade grass) and *Cynodon plectostachyus* (giant star grass). Romosinuano (ROMO; *n* = 308) and Costeño con Cuernos (CCC; *n* = 333) breeds were maintained at the experimental station Turipaná, located in Cereté, Córdoba. ROMO and CCC cattle were fed with a mixture of *Dichantium aristatum* (angleton grass) and *Megathyrsus maximus* (guinea grass). The San Martinero breed (SM; *n* = 415) were maintained at the experimental station La Libertad, located in Villavicencio, Meta and were fed with *Brachiaria decumbens* and *Brachiaria humidicola* (koronivia grass). All animals were given trace mineral salt and water *ad libitum*.

The mating scheme implemented for all breeds included in this study was a cyclical rotational system. In this system, there are a fixed number of families depending on the population size and particular farming management conditions. Initially, bulls from family 1 breed with cows from family 2, bulls from family 2 breed with cows from family 3, and so on. After 3 years, bulls from family 1 breed with cows from family 3, bulls from family 2 breed with cows from family 4, and so on. After another 3 years, bulls from family 1 breed with cows from family 4, bulls from family 2 breed with cows from family 5, and so on. Finally after another 3 years, the initial rotation starts again to complete the cycle.

No cattle tick control methods such as dipping, spraying, vaccination, or any biological or another type of management method were carried out on the animals during the period established for this experiment.

### Metadata

**Fig. 2:**
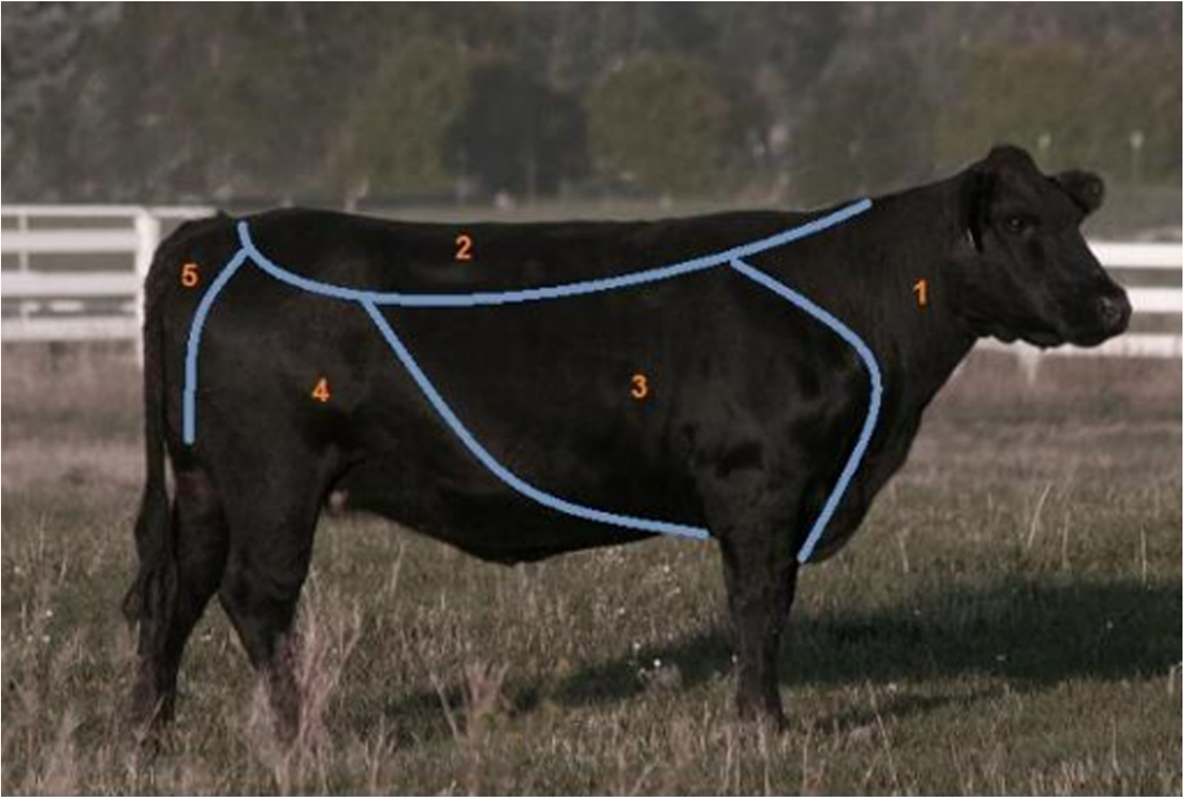
Anatomical zones (Z) differentiated for tick counting. Z1: head and neck; Z2: back and loin; Z3: forelegs, shoulders and ribs; Z4: rear legs, (udder), fore and rear flank; Z5: rump and tail

Cattle were restrained using a squeeze crush and were handled gently in order to minimize stress. Two trained veterinarians performed the tick counting and measured heart and respiratory rates in each experimental station. In total, four samplings were carried out in each cow, which included tick counting and vital sign measurements. All sampling sessions were carried out in the morning, between 7:00 h and 11:00 h. Samplings were carried out between September 2012 and February 2013, with an average of one sampling session per month. Rectal temperature was taken using a digital thermometer, and an infrared thermometer was used to measure body surface temperature. Respiratory rates were measured using a stethoscope. The body of each animal was visually divided into five anatomical zones (Fig. 2) and the counting of ticks was undertaken sequentially as follows: (1) head and neck; (2) back and loin, (3) forelegs, shoulders and ribs; (4) rear legs, (udder), fore and rear flank; (5) rump and tail. No ticks were removed from the animals during counting. Veterinarians that performed the tick counting and vital signs recording were trained and evaluated for the collection of these variables in order to reduce the potential bias.

The breed, family, sex and age of the animals were retrieved from genealogy databases that belong to the Sistema de Bancos de Germoplasma de la Nación para la Alimentación y la Agricultura de Colombia (SBGNAA; System of National Germplasm Banks for Food and Agriculture of Colombia) for animal genetic resources.

### Climatic variables and heat stress indices

Climatic variables were recorded on the same date as tick counting for each of the three experimental stations. Air temperature, dew-point temperature and relative humidity were measured using a data logger device. These variables were taken at the same time when the tick counting, and the vital signs recording were performed on the animals. These parameters were then used to calculate the temperature humidity index (THI) described in [9, 20]:

$$ THI=1.8\times Ta-\left(1- RH\right)\times \left( Ta-14.3\right)+32 $$

where *Ta* is environmental temperature and *RH* is the relative humidity as a fraction of the unit. Average rectal temperature (RT) and respiratory frequency (RF) were calculated for each breed in order to estimate Benezera’s adaptability index (AI) described in [10]. The following formula was used to calculate the AI:

$$ \mathrm{AI}\kern0.28em =\left(\frac{RT}{\overline{RT}}\right){\sigma}_{\mathrm{RT}}+\left(\frac{RF}{\overline{RF}}\right){\sigma}_{\mathrm{RF}} $$

An AI < 2 indicates maximum adaptability while an AI ≥ 2 indicates a state of lower adaptability. After computing these indices for each individual cow, both THI and AI were converted to ordinal variables. Four categories were created for THI (THI < 75; THI ≥ 75 to ≤ 79; THI >79 to ≤ 84; THI > 84) and two for AI (AI ≤ 2; AI > 2).

### Statistical analysis

Statistical analyses were performed using the Statistical Analysis System software v.9.4 (SAS Institute, Cary, North Carolina, USA). Descriptive statistics of the variables were obtained using the MEANS procedure. Four different statistical tests (Shapiro-Wilk, Kolmogorov-Smirnov, Anderson-Darling and Cramér-von Mises) were computed using the UNIVARIATE procedure to estimate parameters describing the distributional properties for the dependent variable (ticks burden). Results confirmed a significant departure from normality and tick burden was analyzed after the logarithmic transformation (base 2) with a linear mixed model using the MIXED procedure. The final model is described below:

$$ {y}_{ijkl m}=\mu +{B}_i+{S}_j+{F}_k\left({B}_i\right)+{N}_l\left({B}_i\right)+{TB}_{mi}+{AB}_{ni}+{b}_1G+{b}_2W+{a}_m+{e}_{ijkl} $$

where *y* is the logarithmic transformation (base 2) of the ticks burden, *μ* is a general mean, *B*_*i*_ is the fixed effect of the *i*^th^ breed, *S*_*j*_ is the fixed effect of the *j*^th^ sex, *F*_*k*_(*B*_*i*_) is the fixed effect of the *k*^th^ family nested in the *i*^th^ breed, *N*_*l*_(*B*_*i*_) is the fixed effect of the *l*^th^ sample date nested in the *i*^th^ breed, *TB*_*mi*_ is the interaction between the *m*^th^ category of the THI index (T) and *B*_*i*_, *AB*_*ni*_ is the interaction between the *n*^th^ category of the adaptability index (A) and *B*_*i*_, *b*_*1*_*G* is the regression coefficient for the covariation of the age at sampling, *b*_*2*_*W* is the regression coefficient for the covariation of live weight at sampling, *a*_*m*_ is the animal random effect, and *e*_*ijkl*_ is the random residual error.

## Results

**Fig. 3:**
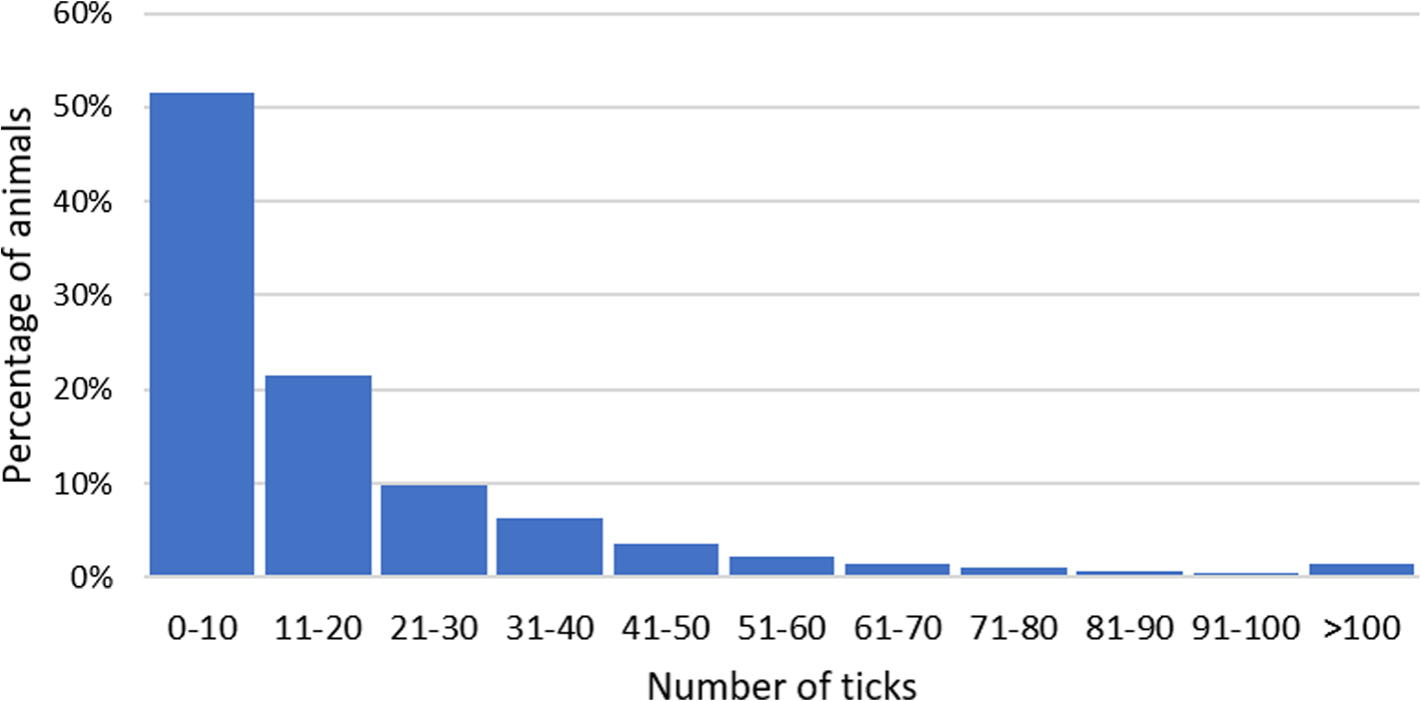
Overall frequency distribution of the percentage of animals per number of tick category for all Colombian cattle breeds

Descriptive statistics of the variables considered in this study are presented in Table 1. The mean live weight was 376 kg with a large variation (range = 585 kg and coefficient of variation = 24.5%). The mean AI was 2.0 (SD = 0.21) and the mean THI was 82.7 (SD = 3.63). The frequency distribution for number of ticks in all breeds is shown in Fig. 3. Frequency distributions were created for each breed; however, the distribution was similar among all four breeds, and therefore only the overall frequency distribution for all breeds is shown.

**Table 1:** Mean, standard deviation (SD), minimum (Min) and maximum (Max) values for age, live weight, temperature-humidity index (THI), adaptability index (AI) and tick burden in four Colombian cattle breeds

Variable	Mean	SD	Min	Max
Age (years)	4.51	2.43	0.80	14.03
Live weight (kg)	375.6	92.1	105.0	690.0
THI	82.66	3.63	66.90	91.63
AI	2.00	0.21	1.39	3.44
Tick burden	17.4	24.6	0	370

**Fig. 4:**
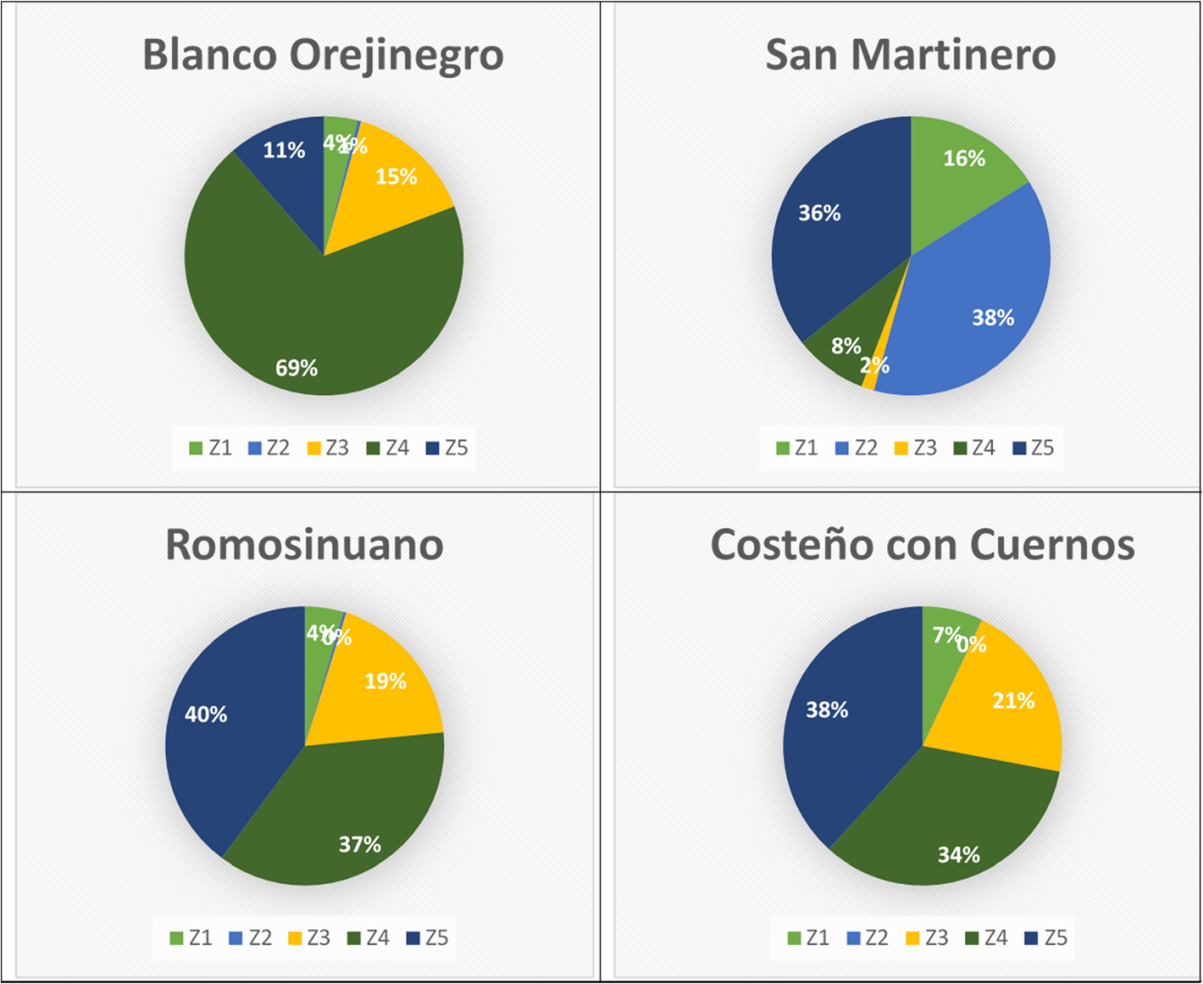
Proportion of average percentage of tick burden by anatomical zone (Z) in four Colombian cattle breeds. *Abbreviations*: Z1, head and neck; Z2, back and loin; Z3, forelegs, shoulders and ribs; Z4, rear legs, (udder), fore and rear flank; Z5, rump and tail

The means and standard deviations of ticks found on each anatomical zone assessed are shown in Table 2 for the four breeds evaluated. The percentage of tick burden by anatomical zone is shown in Fig. 4. In ROMO and CCC cattle, a higher proportion of ticks was observed in the rear legs, udder, fore, rear flank (38–40%), rump and tail (34–37%), while in BON cattle, 69% of the tick burden was observed in the rear legs, udder, fore and rear flank. In the case of SM cattle, most of the ticks were located in the head and neck (16%), in the back and loin (38%), and in the rump and tail (36%).

**Table 2:** Means and standard deviations of tick counts in different body zones in four Colombian cattle breeds

Body zone	Blanco Orejinegro	San Martinero	Romosinuano	Costeño con Cuernos
Z1	0.7 ± 2.5	2.3 ± 5.9	0.6 ± 1.9	2.4 ± 8.7
Z2	0.0 ± 0.3	5.6 ± 8.1	0.0 ± 0.1	0.0 ± 0.1
Z3	2.2 ± 3.8	0.4 ± 1.9	2.5 ± 4.0	4.0 ± 6.4
Z4	10.9 ± 14.4	1.0 ± 2.4	4.6 ± 6.9	7.2 ± 11.1
Z5	2.0 ± 4.0	7.2 ± 12.2	5.8 ± 10.2	9.4 ± 15.1

*Note*: Values shown are means ± standard deviation

*Abbreviations*: Z1, head and neck; Z2, back and loin; Z3, forelegs, shoulders and ribs; Z4, rear legs; (udder), fore and rear flank; Z5, rump and tail

All the effects included in the model had a significant effect on tick burden (Table 3). Males had a lower (*F*_(1,1528)_ = 5.34, *P* = 0.0210) tick burden (18.3 ± 1.9) than females (23.2 ± 1.2). Sample date and family, both nested within breed, were significant on tick load. Likewise, interactions among breed and both AI and THI showed a significant effect on tick burden. Detailed results of these interactions are shown in Table 3 and Table 4. The covariate age (*F*_(1,1528)_ = 5.02, *P* = 0.0253) and live weight (*F*_(1,1528)_ = 16.49, *P* < 0.0001) were also significant. The overall tick burden by breed is shown in Table 4. CCC had the highest tick burden among the four breeds evaluated (31.8 ± 2.3), while ROMO had the lowest tick burden (12.8 ± 2.6). No significant differences for tick burden were found between ROMO and BON breeds (17.7 ± 2.1); however, both ROMO (*t*_(1528)_ = -3.30, *P* = 0.0010) and BON (*t*_(1528)_ = -2.28, *P* = 0.0227) had a significantly lower tick burden than the SM breed (21.0 ± 1.8).

**Table 3:** Significance of the effects included in the model for tick burden in four Colombian cattle breeds

Effect	*df*	*F*-value	*P*-value
Breed	3	9.80	<0.0001
Sex	1	5.34	0.0210
Sample date (nested in breed)	11	27.53	<0.0001
Family (nested in breed)	30	1.73	0.0087
Breed*THI interaction	10	5.11	<0.0001
Breed*AI interaction	4	3.25	0.0115
Age	1	5.02	0.0253
Live weight	1	16.49	<0.0001

*Abbreviations*: THI, temperature-humidity index; AI, adaptability index; df, degrees of freedom

**Table 4:** Tick burden by categories of the adaptability index (means ± standard errors) in four Colombian cattle breeds

	Blanco Orejinegro	San Martinero	Romosinuano	Costeño con Cuernos
Overall	17.7 ± 2.1^a^	21.0 ± 1.8^b^	12.8 ± 2.6^a^	31.8 ± 2.3^c^
AI category				
≤ 2	16.1 ± 2.6^abx^	19.6 ± 2.3^bx^	9.4 ± 3.0^ax^	30.8 ± 2.6^cx^
> 2	19.3 ± 2.2^ax^	22.3 ± 2.1^by^	16.2 ± 2.9^ay^	32.8 ± 2.6^bx^

^ab^Means with different superscripts in the same row are significantly different (*P* < 0.05)

^xy^Means with different superscripts in the same column are significantly different (*P* < 0.05)

**Fig. 5:**
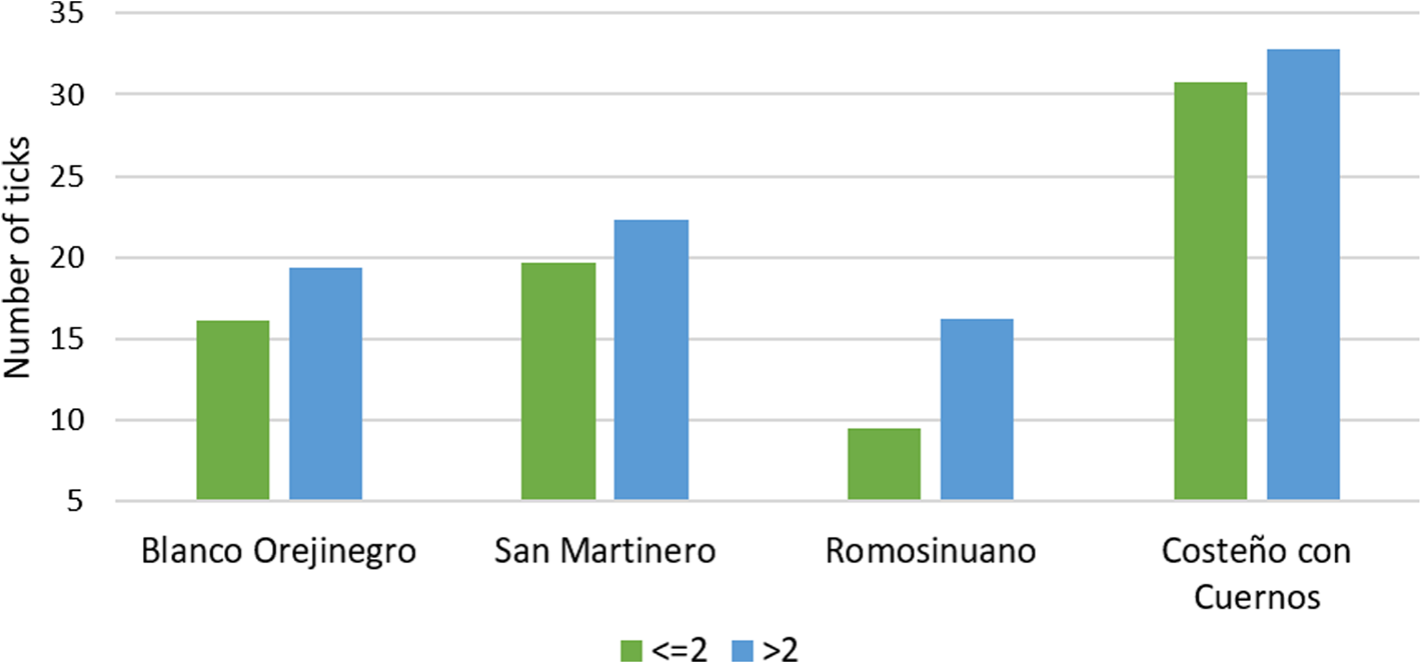
Tick burden by adaptability index (AI) category in four Colombian cattle breeds

An overall effect of AI was observed within breeds (*F*_(4,1528)_ = 3.25, *P* = 0.0115), with higher tick burdens observed when the AI was higher than 2, although this effect was significant only in the SM (*t*_(1528)_ = -2.43, *P* = 0.0153) and ROMO (*t*_(1528)_ = -2.62, *P* = 0.0088) breeds. Tick burden was significantly different between breeds for both AI categories (Table 4 and Fig. 5). When the AI values were lower or equal to 2, CCC individuals showed a higher tick burden than all other breeds (30.8 ± 2.6), and a higher tick burden than ROMO and BON when the AI was higher than 2. Likewise, ROMO had a lower tick burden than SM and CCC breeds in both scenarios (AI lower or higher than 2), while BON showed a lower tick burden compared with SM and CCC only when AI was higher than 2 (19.3 ± 2.2).

**Fig. 6:**
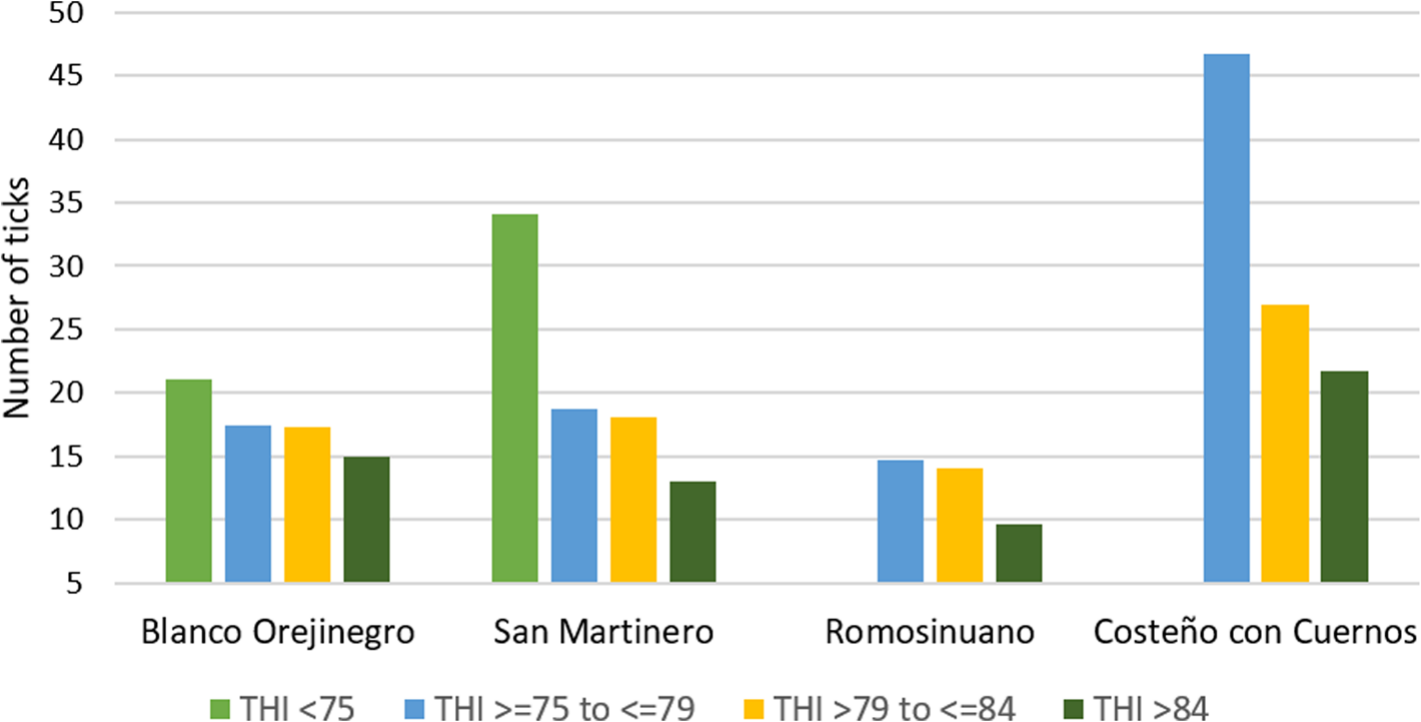
Tick burden by temperature-humidity index (THI) category in four Colombian cattle breeds

There was a significant effect of the interaction between THI and breed on tick burden (*F*_(10,1528)_ = 5.11, *P* < 0.0001), which is illustrated in Table 5. Animals from breeds classified in the same THI category showed different tick burdens. SM animals had a higher (*t*_(1528)_ = -3.62, *P* = 0.0003) tick burden (34.1 ± 5.6) than BON animals (21.1 ± 4.3) when the THI was < 75. However, no differences were found for tick burden between BON, SM and ROMO breeds when the THI was ≥ 75. On the other hand, the tick burden in CCC breed was significantly higher than the other breeds for all the THI categories evaluated. Overall, there was a gradual tick burden decrease in all breeds as the THI value increased (Table 5 and Fig. 6).

**Table 5:** Overall tick burden and tick burden by categories of the temperature-humidity index (means ± standard errors) in four Colombian cattle breeds

THI category	Blanco Orejinegro	San Martinero	Romosinuano	Costeño con Cuernos
< 75	21.1 ± 4.3^ax^	34.1 ± 5.6^bx^	–	–
≥ 75 to ≤ 79	17.5 ± 3.9^ax^	18.8 ± 2.1^ay^	14.7 ± 4.7^ax^	46.7 ± 6.3^bx^
> 79 to ≤ 84	17.3 ± 2.1^ax^	18.1 ± 2.2^ay^	14.0 ± 3.7^ax^	26.9 ± 1.7^by^
> 84	15.0 ± 2.0^ax^	13.0 ± 2.5^az^	9.7 ± 3.9^ay^	21.7 ± 2.3^by^

^abc^Means with different superscripts in the same row are significantly different (*P* < 0.05)

^xy^Means with different superscripts in the same column are significantly different (*P* < 0.05)

## Discussion

There is a scant amount of data in the literature on tick burden in cattle for tropical regions in the Americas. Most studies on this topic have been carried out in subtropical countries such as South Africa [8, 15], countries with extreme high temperatures such as Egypt [16] and Pakistan [17], and some tropical countries such as Tanzania [18] and Nigeria [21]. Tick burden is frequently reported using transformed data, which makes result comparison for actual tick burden difficult to interpret. This study shows the actual tick burden and its relationship with heat stress indices in four *Bos taurus* breeds located in three different geographical regions of Colombia (the Caribbean, Andean and Orinoquia regions). Tick burden in these Colombian cattle breeds was lower than that found in an experiment [19] using steers grazing in the Meta department (Orinoquia region). On the other hand, the average tick burden found in the four Colombian breeds in our experiment (17.4 ± 24.6) is lower than that observed in other tropical regions such as the *B. indicus* Meru crossbred in Tanzania (37.5 ± 11.8) [18] and some *B. indicus* cattle populations (White Fulani and Bunaji breeds and their crossbreds) in Nigeria (22 ± 1.4) [21]. The different cattle breeds, grass species, grazing management, herding system, climate conditions and ecological aspects of ticks might explain the higher tick burden found in these other studies.

### Tick distribution on cattle bodies

The tick burden on the rump and tail of SM (7.2 ± 12.2), ROMO (5.8 ± 10.2) and CCC (9.4 ± 15.1) cattle was higher than the tick burden found on the rear legs, belly and flanks (1.0 ± 2.4, 4.6 ± 6.9 and 7.2 ± 11.1, respectively). These results agree with those obtained by [15] in Nguni cattle. These authors found that the tick burden on tail and perineum was higher than the tick burden on the belly throughout the year. The lowest tick burden in BON, ROMO and CCC cattle was observed on the head and neck followed by the back and loin. Similarly, Asmaa et al. [16] found that the lowest number of ticks were observed around the eyes, neck and chest. Ticks prefer hidden and warm anatomical zones on the animal body [8]. Hence, a suitable environment for tick survival is under the tail and perineum, possibly explaining the high number of ticks observed on these body zones. Likewise, the rear legs, udder, belly, fore and rear flank are more exposed to grass, and therefore easily reached by ticks. This might be the reason why we observed a high proportion of the total tick burden in these body zones.

Different types of grass and paddocks where these animals feed could cause differences in tick burden by anatomical zones. This is corroborated by the similar percentage of tick burden by anatomical zones observed for the ROMO and CCC breeds, which were located at the same experimental station and were fed with the same type of grass. Conversely, the tick burden by anatomical zone is very different in BON and SM breeds, which can be in part explained by a different environment.

### Sex, age and live weight

The higher tick burden observed in females agrees with the results of Rehman et al. [17] in Pakistani cattle, where females had a significantly higher tick burden than males. Likewise, there was a higher prevalence of tick infestation in females (58.7%) than in males (41.5%) in Egyptian cattle [17]. On the other hand, no significant differences were found for the infestation prevalence of *R. microplus* between males and females in Indian cattle [22] and Mexican cattle [23]. In the experimental stations where the cattle for our study were kept, bulls received preferential management since they had resting periods before the start of the next breeding season, and were allocated in paddocks with a lower stocking rate. Conversely, cows were always kept at higher stocking rates. These particular conditions received by bulls during some months of the year might explain why we found a lower tick burden in males compared to females in our study.

We found a significant correlation between age and tick burden in the Colombian breeds evaluated. Several studies have found a significant effect of the age of cattle on tick burden [8, 16, 17, 21]. Rehman et al. [17] and Lorusso et al. [21] found a lower tick burden in calves compared to older cattle, and Asmaa et al. [16] observed a higher infestation rate in older animals of different ruminant livestock species.

Most studies on tick burden in cattle have not evaluated the effect of live weight. Our study showed that in addition to age, live weight also had a significant effect on tick burden: the heavier the animal the higher the tick burden. This agrees with studies where the age had a significant correlation with tick burden in cattle [8, 16, 17, 21] since age and live weight are highly correlated. It is possible to hypothesize that factors contributing to the higher tick burden in heavier animals could be either a compromised immune system in heavier older animals (as they tend to get weaker with age) or that larger animals have a wider skin surface with a denser vasculature.

### Breed and family

The significant effect of breed in our study on tick burden has also been reported in other studies. In South Africa, Marufu et al. [24] found a higher tick burden in Bonsmara heifers (1.8 ± 0.02) compared to Nguni heifers (1.4 ± 0.03). In the latter study, a higher tick infestation rate was found in Friesian cattle (77.5%) compared to the local breeds (50.4%) and also to crosses of Friesian × local breeds (41.8%). Similarly, in Pakistani cattle, Rehman et al. [17] observed the highest tick burden in exotic *Bos taurus* breeds, followed by crossbred and indigenous *Bos indicus* breeds. Asmaa et al. [16] found the highest prevalence of tick infestation in Friesian cattle (77.5%), although in this case the lowest prevalence was not found in local breeds (50.4%) but instead in crossbred animals (41.8%). However, in our study, we did not have tick burden data from other exotic breeds to make these comparisons. Therefore, further research would be required in order to compare the tick burden between Colombian *Bos taurus* cattle breeds and *Bos indicus* breeds, which are widely used on beef cattle farms in the region.

The different environmental conditions found on the experimental stations, where the four Colombian cattle breeds were kept, might have influenced the tick burden on these animals. However, the ROMO and CCC breeds were located in the same experimental station, and they grazed on the same type of grass and received the same herd management. In this case, where we had a greater control of the environment variation, the significant differences found between the two breeds could be attributed to a genetic effect. Moreover, the significant effect of family nested in breed also suggests a possible genetic influence on the individual response to tick attachment. A genetic effect causing the difference in tick burden between ROMO and CCC breeds might be expressed by a particular phenotype such as coat color or hair length. Coat color affected the tick burden of crossbred Gyr × Holstein animals in Brazil [25], which was possibly caused by the higher absorption of heat in animals with darker coat colors. Moreover, the hair length and the level of smoothness of the coat (smooth *vs* woolly) had a significant effect on tick burden in South African cattle [8, 24], while skin thickness significantly affected the tick burden in Brazilian crossbred cattle during dry and rainy seasons [25]. Gene expression studies showed that both immune and non-immune mechanisms are associated with tick resistance in cattle [26]. Differences in tick burden found between ROMO and CCC cattle are less likely to be due to a different coat color or smoothness as these breeds share great similarity in function of these traits. Instead, skin thickness, hair density or the particular immunological response to tick infestation of each breed, might be hypothesized as characteristics with a significant genetic-related effect on the tick burden for these breeds.

### Tick burden and heat stress

Environmental changes have a positive or negative effect on the physiological heat regulation mechanisms of cattle and potentially on the tick burden of these animals [8]. The higher tick burden found in SM and ROMO animals with an AI > 2 would indicate that cattle with a state of lower adaptability, expressed by the experience of a thermal discomfort, have a higher susceptibility to be infested by ticks. The better thermal adaptation of some animals (with an AI < 2) might be explained by having a particular phenotype for some of the traits discussed above (shorter hair, lighter/brighter coat, smooth coat). However, the ability of cattle to dissipate heat load by sweating and panting is compromised in hot and humid conditions making cattle experience heat stress much faster than other livestock species such as swine [9]. Therefore, the aforementioned adaptation-related traits would be particularly important for cattle located in the tropics, since humidity levels are higher in this region throughout the whole year compared with other latitudes.

The tick burden in cattle might be affected by the same traits that have an influence on the thermal comfort of animals. For instance, cattle with longer hair or a woolly coat would struggle to dissipate heat, but at the same time, this phenotype would make the attachment of ticks easier to the body. In the case of coat color, some studies found higher tick burdens in cattle with a dark coat compared with those that had lighter coat colors [23, 25], which might be due to the higher heat absorbance by darker coats, making the skin warmer and more attractive for parasites. On the other hand, traits such as skin thickness, hair density and skin secretions could have a role for tick resistance in domestic livestock [27]. These latter traits might also have an influence on the cattle thermal comfort since they affect the ability of the animal to dissipate heat. Further studies on ticks in Colombian cattle breeds should include variables such as hair length, coat color and skin characteristics in order to analyze its influence on tick infestation levels and determine possible significant differences for these traits among breeds.

The relationship observed between THI and tick burden indicates that high THI values would be associated with a lower tick burden on the breeds considered in our study. On the other hand, and according to the results on AI described in our study, a higher tick infestation would be expected when animals experience a higher thermal discomfort. However, THI does not account for variables taken directly from cattle but instead this index measures the level of thermal stress by accounting for the effects of air temperature and humidity in the environment [9]. The tick burden on cattle is related with the survival of these parasites in the environment. In Australia, Sutherst et al. [28] found that the egg production of *Boophilus microplus* was negatively affected by soil temperatures greater than 33.6 °C or by a soil moisture less than 0.26 water-holding capacity when the soil maximum temperature was greater than 37.2 °C. Likewise, the authors also found a reduced tick egg survival with a soil temperature greater than 25.4 °C and soil moisture below 0.21 water-holding capacity. It is possible that under tropical conditions, the viability of tick eggs or the survival of the tick itself is compromised when temperatures reach a certain threshold. Furthermore, high environmental temperatures can affect the hair growth in cattle so that shorter and fewer hairs on the body would allow animals to lose more heat from sweating and evaporation when temperatures are elevated [8], and at the same time, it would discourage tick attachment to the skin.

Results from our study suggest that the THI threshold at which the tick burden on cattle is affected would differ depending on the breed. For instance, the tick burden in SM was lower when THI was greater than 75, and the lowest tick burden was observed with a THI greater than 84. On the other hand, the two breeds studied in the Caribbean region showed different THI thresholds. While the CCC breed showed a lower tick burden when THI was greater than 79, a tick burden reduction in ROMO was only significant when THI was greater than 84. This would indicate that although there might be a decrease of the tick eggs viability/tick survival as heat stress increases in the environment, the individual genetic resistance of cattle would set the infestation level that they can tolerate under different heat-stress conditions. The present study also showed a significant correlation between both breed and family with the tick burden of Colombian cattle breeds. This could have potential implications for selection and mating decisions within breeding programs to obtain future generations of pure or crossbred cattle with higher tick resistance. On the other hand, the significant correlation of both age and sex with tick burden is key information that might be eventually used to improve herding strategies and to develop new tick control methods. The association between heat stress and tick burden on cattle would suggest that the implementation of grazing and management strategies to minimize heat stress might be used as a method to reduce the tick burden in cattle herds.

## Conclusions

There was a significant association of breed, sex, age and live weight on the tick burden of *Bos taurus* Colombian cattle breeds. The particular environmental conditions of each agroecosystem might have affected the differences observed in the distribution of ticks on the body of cattle. A higher thermal discomfort is associated with a higher tick burden in cattle that possibly have a lower level of adaptability, and this relationship would be directly affected by some of the traits that have an influence on the thermal comfort of the animal. A decrease of the tick burden in cattle could be associated with higher heat stress levels in a tropical environment. However, this interaction between tick burden and environmental heat stress might be affected by characteristics of the agroecological region itself, the breed, the genetic resistance of the individual tick and the thermal adaptability of cattle.

## Abbreviations

ROMO: Romosinuano
CCC: Costeño con Cuernos
SM: San Martinero
BON: Blanco Orejinegro
AI: Adaptability index
THI: Temperature humidity index

## References

[1] Jacobs D, Fox M, Gibbons L, Hermosilla C. Principles of Veterinary Parasitology. Chichester, UK: Wiley Blackwell; 2016.

[2] Taylor MA, Coop RL, Wall RL.Veterinary Parasitology. Chichester, UK: Wiley Blackwell; 2016.

[3] Jonsson NN, Davis R, De Witt M (2001). An estimate of the economic effects of cattle tick (*Boophilus microplus*) infestation on Queensland dairy farms. Aust Vet J..

[4] Polanco DN, Ríos LA (2016). Biological and ecological aspects of hard ticks. Corpoica Cien Tecnol Agropecuaria..

[5] Cortés JA, Betancourt JA, Argüelles J, Pulido LA (2010). Distribution of *Rhipicephalus* (*Boophilus*) *microplus* ticks on cattle and farms from altiplano cundiboyacense (Colombia). Corpoica Cien Tecnol Agropecuaria..

[6] Jonsson NN (2006). The productivity effects of cattle tick (*Boophilus microplus*) infestation on cattle, with particular reference to *Bos indicus* cattle and their crosses. Vet Parasitol..

[7] Estrada-Peña A, Acedo CS, Quílez J, Del Cacho E (2005). A retrospective study of climatic suitability for the tick *Rhipicephalus* (*Boophilus*) *microplus* in the Americas. Global Ecol Biogeogr..

[8] Katiyatiya CLF, Muchenje V, Mushunje A (2014). Seasonal variation in coat characteristics, tick loads, cortisol levels, some physiological parameters and temperature humidity index on Nguni cows raised in low- and high-input farms. Int J Biometeorol..

[9] Bohmanova J, Misztal I, Cole JB (2007). Temperature-humidity indices as indicators of milk production losses due to heat stress. J Dairy Sci..

[10] Benezra MV (1954). A new index for measuring the adaptability of cattle to tropical conditions. J Anim Sci..

[11] Rhoad AO (1944). The Iberia heat tolerance test for cattle. Trop Agr..

[12] Rocha JF, Gallego JL, Vasquez RF, Pedraza JA, Echeverri J, Cerón-Muñoz MF, Martínez RA (2012). Estimation of genetic parameters for age at first calving and calving interval in Blanco Orejinegro (BON) breed cattle populations in Colombia. Rev Colomb Cienc Pecuarias..

[13] Martínez R, Quiceno J, Gallego J, Mateus H, Rodríguez O, Medina P, Ballesteros H (2012). Growth performance of Blanco Orejinegro and Romosinuano bullocks on pasture. Rev Col Cienc Pec..

[14] Martínez R, Dunner S, Toro R, Tobón J, Gallego J, Cañón J (2010). Effect of polymorphisms in the Slc11a1 coding region on resistance to brucellosis by macrophages *in vitro* and after challenge in two *Bos* breeds (Blanco Orejinegro and Zebu). Genet Mol Biol..

[15] Mapholi NO, Maiwashe A, Matika O, Riggio V, Banga C, MacNeil MD (2017). Genetic parameters for tick counts across months for different tick species and anatomical locations in South African Nguni cattle. Trop Anim Health Prod..

[16] Asmaa NM, ElBably MA, Shokier KA (2014). Studies on prevalence, risk indicators and control options for tick infestation in ruminants. Beni-Suef University J Basic Appl Sci..

[17] Rehman A, Nijhof AM, Sauter-Louis C, Schauer B, Staubach C, Conraths FJ (2017). Distribution of ticks infesting ruminants and risk factors associated with high tick prevalence in livestock farms in the semi-arid and arid agro-ecological zones of Pakistan. Parasit Vectors..

[18] Wambura PN, Gwakisa PS, Silayo RS, Rugaimukamu EA (1998). Breed-associated resistance to tick infestation in *Bos indicus* and their crosses with *Bos taurus*. Vet Parasitol..

[19] Aycardi E, Benavides E, Garcia O, Mateus G, Henao F, Zuluaga FN (1984). *Boophilus microplus* tick burdens on grazing cattle in Colombia. Trop Anim Health Prod..

[20] CIP (2006). Livestock Feeding Strategies Simulation Models.

[21] Lorusso V, Picozzi K, de Bronsvoort B, Majekodunmi A, Dongkum C, Balak G (2013). Ixodid ticks of traditionally managed cattle in central Nigeria: where *Rhipicephalus* (*Boophilus*) *microplus* does not dare (yet?). Parasite Vector.

[22] Singh NK, Rath SS (2013). Epidemiology of ixodid ticks in cattle population of various agro-climatic zones of Punjab, India. Asian Pacific J Trop Med..

[23] Rodríguez-Gallegos CE, Acosta-Rodríguez MR (2011). Genetic and environmental factors influencing the resistance of terminal cross calves to tick *Rhipicephalus* (*Boophilus*) *microplus* and horn fly *Haematobia irritans*. Trop Subtrop Agroecosyst..

[24] Marufu MC, Qokweni L, Chimonyo M, Dzama K (2011). Relationships between tick counts and coat characteristics in Nguni and Bonsmara cattle reared on semiarid rangelands in South Africa. Ticks Tick-Borne Dis..

[25] Machado MA, S Azevedo AL, Teodoro RL, Pires MA, Cd Peixoto MG, de Freitas C (2010). Genome wide scan for quantitative trait loci affecting tick resistance in cattle (*Bos taurus × Bos indicus*). BMC Genomics..

[26] Porto Neto LR, Jonsson NN, D’Occhio MJ, Barendse W (2011). Molecular genetic approaches for identifying the basis of variation in resistance to tick infestation in cattle. Vet Parasitol..

[27] Shyma KP, Gupta JP, Singh V (2015). Breeding strategies for tick resistance in tropical cattle: a sustainable approach for tick control. J Parasit Dis..

[28] Sutherst RW, Bourne AS, Sutherland ID (1999). Production and survival of eggs of the cattle tick *Boophilus microplus* (Canestrini) (Acarina: Ixodidae) in the wet and dry tropics of north Queensland. Aus J Entomol..

